# The Brownhill Case at Birmingham

**Published:** 1911-12-16

**Authors:** Howard J. Collins

**Affiliations:** House Governor. General Hospital, Birmingham


### The Editor's Letter Box.

### THE BROWNHILL CASE AT BIRMINGHAM.

*To the Editor of* The Hospital.

Sir,—In your comment on the above case, you have entirely omitted the original complaint made by the husband of the patient on the Saturday morning, that she was not allowed to go into the casualty-room out of her turn for her hand to be re-dressed, in reply to which it was pointed out that as there were one hundred patients or more who had come at nine o'clock they must be seen in preference to one that came at 9.45. A complaint was then made about the waiting on the Thursday on her first visit, into which the House Governor undertook to make inquiries. On the Monday morning the rudeness to the doctors took place, and after her hand was dressed she was told not to attend again in consequence of this. With regard to the occurrence on Wednesday, the House Governor saw both the woman and her husband, and again repeated that if she would express her regret for her rudeness she would be treated again, but otherwise should not attend. As a matter of fact, she has not expressed any apology, promptly or otherwise; but on her statement that she had no intention to be rude the House Governor arranged for her to be seen on the Friday morning by the senior officer on duty in the department, and she is now being treated as often as her injury requires.

The statements, therefore, that she "had apologised to the doctors and been refused" or that "they had continued to refuse treatment" are quite untrue.—Yours faithfully, Howard J. Collins,

General Hospital, Birmingham, House Governor.

December 6.

